# Histological transformation in lung adenocarcinoma: Insights of mechanisms and therapeutic windows

**DOI:** 10.1515/jtim-2024-0019

**Published:** 2024-11-06

**Authors:** Nuopei Tan, Yan Li, Jianming Ying, Wanqing Chen

**Affiliations:** Office of Cancer Screening, National Cancer Center/National Clinical Research Center for Cancer/Cancer Hospital, Chinese Academy of Medical Sciences and Peking Union Medical College, Beijing, China; Department of Pathology, National Cancer Center/National Clinical Research Center for Cancer/Cancer Hospital, Chinese Academy of Medical Sciences and Peking Union Medical College, Beijing, China

**Keywords:** non-small cell lung carcinoma, acquired resistance, histological transformation

## Abstract

Histological transformation from lung adenocarcinoma (ADC) to small cell lung carcinoma (SCLC), large cell neuroendocrine carcinoma (LCNEC), squamous cell carcinoma (SCC), and sarcomatoid carcinoma (PSC) after targeted therapies is recognized as a mechanism of resistance in ADC treatments. Patients with transformed lung cancer typically experience a poor prognosis and short survival time. However, effective treatment options for these patients are currently lacking. Therefore, understanding the mechanisms underlying histological transformation is crucial for the development of effective therapies. Hypotheses including intra-tumoral heterogeneity, cancer stem cells, and alteration of suppressor genes have been proposed to explain the mechanism of histological transformation. In this review, we provide a comprehensive overview of the known molecular features and signaling pathways of transformed tumors, and summarized potential therapies based on previous findings.

## Introduction

Lung cancer is the leading cause of cancer related deaths worldwide.^[1]^ Non-small cell lung carcinoma (NSCLC) and small cell lung cancer (SCLC) are the two main histological types, accounting for approximately 80% and 20% of lung cancer cases, respectively. NSCLC can be further classified into three major subtypes: lung adenocarcinoma (ADC), squamous cell carcinoma (SCC), and large-cell carcinoma, among which ADC is the most common subtypes.^[1]^ The transformation of ADC to other histological subtypes has been identified as a significant mechanism of resistance in patients after treatment with tyrosine kinase inhibitors (TKIs) and chemotherapy.^[2,3]^ Pre-existing tumor heterogeneity can lead to histological transformation.^[5,6]^ Dysregulation of signaling pathways in cancer stem cells may also contribute to generate transformed histology.^[7,8,9]^ Additionally, efforts have been made to evaluate the effect of inactivating suppressor genes on ADC transformation.^[10]^ However, the precise mechanisms underlying histological transformation remain unclear as insufficient evidence and a lack of systematic summarization.

Patients with transformed lung cancer typically have poor prognosis and limited response to treatments.^[2,11]^ Despite receiving another treatments after histological transformation, patients still tend to have a short survival time.^[12]^ Summarizations of cases involving histological transformation post-treatment have revealed that more than two-thirds of patients develop histological transformation after developing resistance to immunotherapy. Additionally, it is likely that the real-world frequency of adenocarcinoma transitioning to other subtypes is underestimated, presenting a challenge to clinicians. Unfortunately, guidelines for these patients have not clearly defined any molecular targets for treatment, leaving chemotherapy as the sole option, albeit with poor response rates.^[3]^

Biomarkers are gradually being recognized as significant predictors of histological transformation. Several studies characterized the features of lung cancer histological transformation by next generation sequencing and other potentially detective tools.^[13,14,15,16]^ Therefore, understanding the specific molecular features and signaling pathways of transformed tumors can be helpful to find novel therapies.

Overall, there is clinical need to conclude the previous studies of histologic transformation. Thus, we summarized mechanisms underlying NSCLC transformation and discussed potential treatments for four different transformed tumors.

## Potential mechanisms for tumor histological transformation after treatments

### Intra-tumoral heterogeneity and clonal evolution

Intra-tumor heterogeneity, which is characterized by genetic or epigenetic alterations during spatiotemporal clonal evolution, contributes to the non-uniform distribution of various subclones.^[6,17]^ High intratumoural heterogeneity in tumors may lead to inferior clinical outcomes in patients, as this heterogeneity can contribute to the emergence of treatment resistance under therapeutic selective pressure, either through the expansion of pre-existing subclonal populations or the development of drug-tolerant cells.^[18]^ Cellular heterogeneity in mixed tumors, such as the combination of SCLC and ADC, reveals intriguing similarities in differentiation while representing distinct tumor types. It is plausible to assume that different histological subtypes, such as ADC and SCLC, may coexist within the same initial tumor, with the non-resistant subtype (SCLC) becoming dominant after an initial response to selective drugs.^[19,20,21]^ However, several cases were contradicted to this hypothesis. These studies involved patients with coexisting lung cancer have shown prolonged response to TKIs, which were not observed in patients with a less response to EGFR inhibitors and acquired resistance at an earlier time. These patients exhibited greater tumor growth at the time of SCLC diagnosis.^[21,22]^ Additionally, a case study thoroughly detected initial ADC samples for transformed SCLC and ruled out the possibility of coexisting ADC and squamous cell carcinoma.^[23]^

From an evolutionary perspective, various theories have been proposed to understand the progression of tumors and the factors contributing to treatment failure. In 1976, Nowell introduced the theory of clonal evolution, which applied evolutionary models to investigate tumor development. According to this theory, a tumor originates from a single cell and subsequently undergoes clonal expansion, resulting in a population of cancer cells. Throughout the process of tumorigenesis and evolution, cancer cells experience different levels of genetic instability, leading to the acquisition of diverse genetic abnormalities and subsequent tumor heterogeneity due to additional mutations.^[24,25]^ Quite a few EGFR-mutant tumors conserved the original EGFR mutation despite switching from NSCLC to SCLC.^[26]^ This observation supports the clonal evolution hypothesis.^[27]^ However, the question remains whether SCLC is derived from pre-transformed ADC through linear evolution or if it shares a common precursor cell with ADC through branched evolution.

#### Linear evolution hypothesis

In the clonal evolution model, linear mutation refers to the gradual replacement of the original clone cells by more competitive and aggressive clone cells. Conversely, a case study had opposite results. After treated with chemotherapy combined with TKIs, the transformed SCC only remained the original EGFR mutation and lacked EGFR amplification which existed in the initial ADC. The researchers denied the hypothesis that EGFR amplification was eliminated by treatments. Instead, they proposed that non-amplified colon existed within the tumor initially and survived after the selective pressure exerted by treatments, subsequently progressing to SCLC.^[28]^ It is worth noting that a previous study provided evidence suggesting that linear evolution emphasizes the role of a single gene rather than the entire genome, thereby underestimating the significance of clonal diversity.^[29]^

#### Branching evolution hypothesis

**Figure 1:**
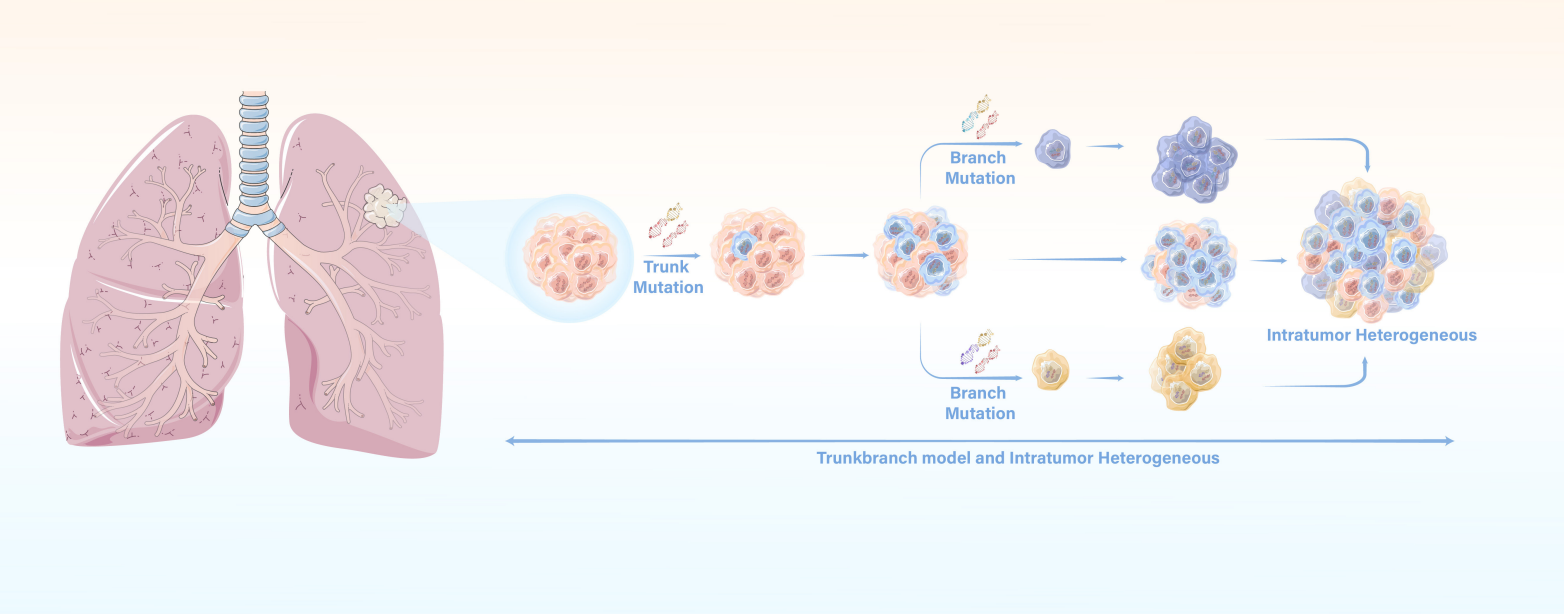
The schematic diagram of Trunk-branch model and Intra-tumor heterogeneity.

Breakthroughs in emerging sequencing technologies revealed a complexity that was previously underestimated.^[30]^ Two previous studies applied multiregional sequencing to analyze surgically resected lung cancers, providing valuable insights into clonal evaluation in NSCLC.^[13,31]^ These investigations supported the concept of branching evolution, also known as the trunk-branch model (Figure 1), which involves the accumulation of driver mutations in subclonal populations.^[32]^ Progenitor clone cells carried early somatic alterations that initiated tumorigenesis and were found throughout all tumor regions. Most known driver mutations were detected in all regions of the same tumor.^[33,34]^ On the other hand, branch mutations contributed to heterogeneity and were present only in specific tumor regions, while private branch mutations were exclusive to a single region.^[13,31]^ These diverse mutations highlighted the sequential mutational evolution of tumors, with trunk mutations occurring early and branch mutations arising at later stages of tumor progression.^[13,31]^ These studies confirmed the presence of intra-tumoral heterogeneity resulting from branch or private mutations, both of which drive tumor progression. Consistent with the aforementioned studies, a perspective cohort study also proved that intratumor heterogeneity and branched evolution were universal phenomenon in ADC.^[35]^ These groups also discovered exposure to tobacco or other chemicals as important features related to numbers of subclonal mutations. They hypothesized that apolipoprotein B mRNA editing enzyme catalytic polypeptide (APOBEC) mutagenesis might contribute to induce subclonal expansions. Additionally, Lee *et al*. shed light on the private evolution of SCLC precursors. They defined the less driven-oncology proliferation cells after being treated with TKIs as persister, which can be induced by APOBEC and subsequently progressed to SCLC.^[36]^ This finding suggested that targeting the enzymatic activity of APOBEC may limit subclone diversification.

In the context of lung cancer, clonal evolution acts as a fundamental mechanism that underlies intratumor heterogeneity. A pivotal framework for comprehending this heterogeneity is the branching evolution model, which offers a more compelling explanation compared to the linear evolution hypothesis. This model elucidates the sequential mutational evolution, wherein initial NSCLC samples harbor diverse subclones. Some of these subclones are eliminated by treatment, while others survive and contribute to the emergence of distinct histological subtypes of lung cancer. In essence, the branching evolution model provides a more nuanced understanding of how intratumor heterogeneity arises and evolves in lung cancer, which is crucial for developing effective, personalized treatment strategies.

#### Detection for tumor heterogeneity

Single-site biopsy sampling has limitations in accurately assessing tumor heterogeneity due to the location of progressing lesions and the intervals between samplings tolerated by the patient. Although circulating cell-free tumor DNA (ctDNA) has been successfully used to identify tumor-specific abnormalities, the role of complementary modalities in detecting histological transformation remains uncertain.^[18,37]^ A newly developed noninvasive radiomics methodology can be used to assess imaging intratumor heterogeneity (IITH) through imaging, which has been shown to predict prognosis in breast cancer. The researchers also identified ferroptosis as a potential therapeutic target for high IITH tumors.^[38]^ Therefore, there is an urgent need for novel approaches such as multiregion and temporal-dynamic sequencing to dissect the complex clonal architecture of lung cancers, which may contribute to predicting histological transformation.

### Cancer stem cells

Cancer stem cells (CSCs) has been shown to contribute to tumor recurrence and metastasis, posing challenges in the treatment of lung cancer.^[39,40]^ These cells possess intrinsic self-renewal and tumorigenic properties, enabling them to resist anti-cancer treatments.^[10]^ Dysregulation of signaling pathways in CSCs leads to abnormal expression of tumor molecular markers, promoting lung tumorigenesis and resistance to chemotherapy.^[7,41]^ It has been proposed that the overexpression of specific molecules in lung CSCs may lead to the development of various histological types of lung tumors which can induce drug resistance in clinical settings.^[8,9,42]^

#### Alveolar type II as CSCs of ADC and neuroendocrine cell

**Figure 2:**
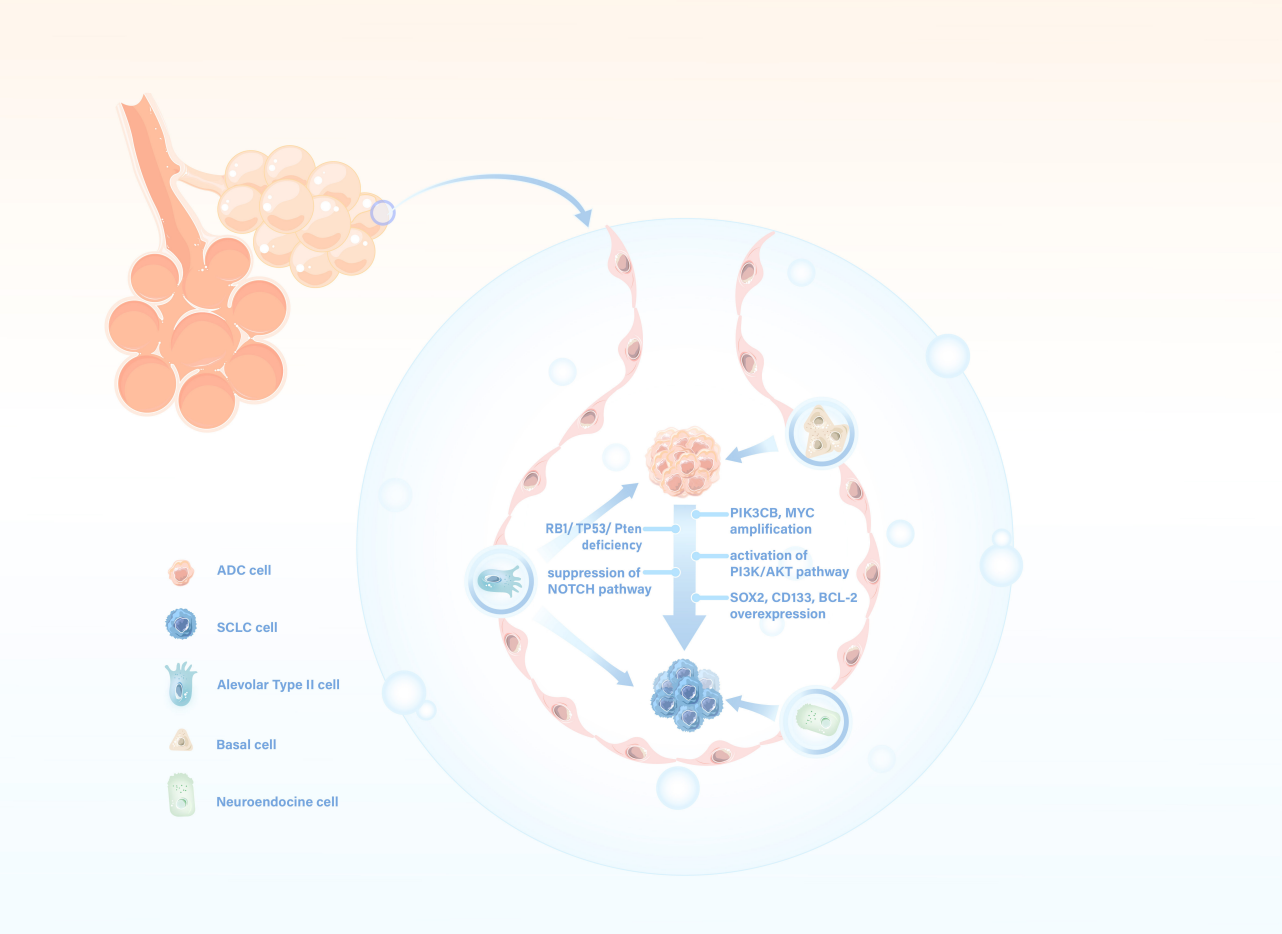
Cancer stem cell theory and potential mechanisms underlying the transformation from ADC to SCLC. ADC: lung adenocarcinoma; SCLC: small cell lung carcinoma.

It is widely accepted that SCLC originates from neuroendocrine cell, while ADC originates from alveolar type II (AT2).^[43]^ ADC cells at various histologic stages exhibit similarities to AT2 cells, particularly during atypical adenomatous hyperplasia. These cells appear to undergo a dedifferentiation process, adopting a stem-like phenotype that is pivotal for the initiation and perpetuation of tumor progression (Figure 2).^[8]^ Subsequent studies revealed higher expression levels of *MYC* and *n-MYC* in CSC-like populations derived from NSCLC, including SCC and ADC, compared to that in ADC-derived cell lines.^[9]^ Additionally, recent *in vitro* studies have observed that the transformation from ADC to SCC can be induced by the overexpression of p40, a squamous marker, either through the combined overexpression of MYC and myrAKT or by MYC alone, albeit at reduced levels, underscoring its significant role as a driver of stemness and histological transformation.^[42]^

#### CD133 expressing CSCs for NSCLC and SCLC

Several studies have identified CD133 cells as CSC that express several stemness genes.^[44]^ CD133 is widely distributed in NSCLC cells at low levels but becomes overexpressed and enriched after treatment with cisplatin.^[45]^ CD133^-^ expressing SCLC cells have also demonstrated resistance to chemotherapy.^[46]^ Sarvi *et al*. isolated CD133 cells from SCLC cell lines and confirmed their CSC characteristics.^[46]^ After treated with chemotherapy, an increased frequency of CD133 expression was noted in cells derived from SCLC specimens.^[46]^ Notably, SCLC-derived CD133 cells showed greater capacity to initiate tumors in nude mice.^[47]^ These studies further revealed that neuropeptide receptors in SCLC-derived CD133 cells and Achaete-scute homolog-1 (ASCL1), a transcription factor in differentiated lung neuroendocrine cells, are essential for CD133 expression in SCLCs.^[46,47]^ Collectively, these findings suggest a potential stem cell theory that significantly contributes to the histological transformation in subtypes of lung cancer. Further studies are warranted to confirm these observations and explore the downstream signaling pathways involved in this model.

#### Biomarkers of CSCs

Fluorescence-activated cell sorting, combined with specific antibodies binding to CSC-surface markers, was applied to conduct a comprehensive analysis of CSCs. The accurate assessment of CSC biomarkers is crucial for the development of effective therapies. Elevated expressions of various markers such as CD133, CD44, and Nestin, which are associated with increased resistance to chemotherapy, tumor-initiating capacities, and sphere formation in adenocarcinoma (AC)-derived cells, have been observed in stemness cells within NSCLC compared to ADC cell lines.^[9,48,49,50]^ Additionally, high levels of acetaldehyde dehydrogenase (ALDH) have been identified as a key CSC surface marker and exhibit a significant correlation with cancer stemness and the immune landscape, as indicated by immune cell counts. It is worth noting that immune-related gene-prognostic models based on the stemness index (mRNAs) could potentially serve as biomarkers for ADC and SCC.^[51]^ The application of these biomarkers is vital to ensure that patients receive timely and appropriate pharmacological interventions.

### Inactivation of tumor suppressor genes

#### *TP53* and *RB1* gene

Prior research has highlighted that baseline *TP53* and *RB1* mutations may differentiate ADC patients at risk for SCLC transformation (Figure 2).^[10]^ TP53 loss was observed in tumor biopsies from 37 patients with drug-resistant NSCLCs carrying EGFR mutations.^[14]^ Analysis of tumor samples and cell lines from EGFR mutant patients resistant to treatment showed that *Rb loss* occurs in all cases that have transformed into SCLC, while it is rare in those that maintain NSCLC characteristics.^[52]^ An *in vivo* study utilized adenoviral vectors targeting Cre recombinase to three distinct cell types to investigate the effect of *TP53* and *RB1* inactivation. The findings indicated that AT2 cells, upon losing *TP53* and *RB1*, exhibited neuroendocrine differentiation traits, akin to the progenitor cells of SCLC.^[10]^ This supports the notion that AT2 cells could be the cellular origin for both ADC and SCLC. Complete inactivation of both *TP53* and *RB1* is associated with an increased risk of SCLC transformation and can be detected by immunohistochemistry (IHC) in the early stages of ADC, consistent with previous reports by Lee *et al*.^[36,53]^ Furthermore, the absence of *Rb1* and *TP53* expression by IHC has been observed not only in the SCLC transformation component but also in large cell neuroendocrine carcinoma (LCNEC) that has transitioned from ADC, potentially under the selective pressure of EGFR-TKI treatment.^[54,55]^

#### *Pten* gene

Recently, an experiment reconstructed pulmonary histological transformation in a mouse model and discovered that the loss of a suppressor gene named *Pten* was related to transformation from ADC to SCLC.^[56]^ The results demonstrated that AT2 cells are refractory to transformation by the low level of Myc expression, which was restricted by *Pten*. The deletion of *Pten* allowed Myc to transform the AT2 lineage, and the additional loss of Rb1 was required for transformation to a neuroendocrine phenotype, illustrating a synergistic interaction that underscores the multi-step nature of tumorigenesis in lung cancers.^[56]^ The researchers also observed that *Rb1* loss constitutes a necessary element for the ADC to SCLC transition, it alone does not result in a fully penetrant transformation. The collaboration between *Rb1* loss and *Myc* expression accelerates neuroendocrine transformation, indicating that both *Myc* and *Rb1* play crucial roles as regulators of neuroendocrine high transformation.^[56]^

#### Live kinase B1 gene

Deletion of *LKB1* not only promotes the occurrence and progression of lung cancer but also specifically leads to tumor heterogeneity, resulting in the development of ADC, SCC, and adenosquamous carcinoma and leading to drug resistance.^[57]^ A previous study utilizing a mouse model with Kirsten rat sarcoma 2 viral oncogene homolog (KRAS) mutant lung cancers indicated that *LKB1*, also known as serine/threonine kinase (STK11), plays a significant role in the gradual transition and poor efficacy of TKIs through metabolic alterations.^[58]^ This was further supported by the following observation *in vivo* that demonstrated accelerated malignant progression and metastasis of *LKB1* loss in lung ADC to SCC transformation.^[59]^ These insights suggested that tumor suppressor proteins could provide a potential explanation for understanding the histological transformation processes in lung cancer patients.

It is worth noting that a case study found that the transformed SCC component exhibited different morphological features compared to the original ADC within the same tumor but shared the same mutations.^[60]^ Subsequent studies bore these findings out and suggested that morphology may not be primarily determined by genomic features.^[61,62]^ In summary, ADC histological transformation after treatments may be influenced by both genomic and transcriptomic alterations.

## Histological transformation in treated ADC

### SCLC transformation

SCLC transformation has been identified as a resistance mechanism in 4%–14% of cases of EGFR-TKIs relapsed ADC.^[14,63]^ Genomic sequencing of EGFR in transformed SCLC tumor samples revealed the preservation of the original EGFR-activating mutation, indicating that these transformed tumors were not de-novo cancers but rather a phenotypic change in response to treatment.^[14]^ This suggested that the SCLC component becomes dominant after successful treatment with EGFR TKIs targeting the ADC component. However, clinical observations have contradicted this hypothesis, as most patients have shown a good response to EGFR-TKIs for extended periods, and greater tumor growth has been observed at the time of SCLC diagnosis. These observations cannot be explained solely by tumor heterogeneity.^[14]^ One possible explanation for these findings is the difference in doubling time between SCLC and ADC. SCLC has a shorter doubling time compared to ADC,^[64]^ which suggests that patients with SCLC transformation would be more likely to develop resistance earlier after EGFR-TKI treatment. ^16, 52^ Further research is needed to validate this hypothesis.

ADC with non-EGFR mutations also has the potential for SCLC transformation, albeit with a longer time to transformation compared to those with EGFR mutations. A case report described a patient with anaplastic lymphoma kinase mutation (*ALK*) rearrangement who experienced SCLC transformation. The original specimen was identified as poorly differentiated *ALK*-fusion ADC.^[65]^ The author proposed that the tumor morphological changes were a result of alectinib treatment rather than an independent de novo cancer.^[65]^ This finding aligns with previous studies reporting SCLC transformation in *ALK*-fusion NSCLC patients after treatment with *ALK* inhibitors.^[65,66]^ Interestingly, a retrospective study revealed a longer median time to SCLC transformation in the non-EGFR mutation group compared to the EGFR-mutant group (26 months versus 16 months). Moreover, individuals with EGFR mutations were found to be at a higher risk of SCLC transformation.^[26,67]^ The different transformation paths may be influenced by various exogenous selective pressures. Overall, SCLC transformation is recognized as a prevalent mechanism of resistance to TKIs in NSCLC, a resistant mechanism for immunotherapy in SCC, LCNEC, and poorly differentiated NSCLC.^[68]^ However, with limited rebiopsy performed after immunotherapy, the frequency of SCLC transformation with immunotherapy is probably underestimated. More research and rebiopsy studies are needed to better understand the occurrence and impact of SCLC transformation in the context of various treatments.

Previous studies hypothesized that histology in lung tumor was not primarily determined by genomic factors. Tang *et al*. conducted a study using whole-exome sequencing and microarray profiling on mixed lung tumor histology samples from 9 patients. The results proved that different histological components within the same tumor shared majority of mutations, a similar mutational spectrum, mutational signatures, the same somatic copy number aberration profiles and similar subclonal architecture.^[62]^ However, the same histology in different lung cancer patients did not share the same mutational signatures, which would be expected to be enriched in specific histology. The researchers considered that somatic mutations may not be the primary determinant of histology, as many genomic characteristics occurred prior to the late events of tumor evolution, such as the separation of different subclones that generate various lung cancer histologies.^[62]^ The team further investigated the relationship between transcriptomic patterns and histological subtypes. The results showed that the gene expression profiles in the same histological components from different patients appeared to be more similar than those in different histological components from the same tumor.^[62]^ This finding suggests the importance of transcriptomic levels in understanding mixed lung tumor histology. Another study focused on ADC to SCLC transformation and supported the aforementioned conclusions.^[61]^ In this study, comprehensive multiomic characterization of ADC to SCLC transformation was performed, revealing similar patterns of shifting signaling pathways from pretransformation ADC to transformed SCLC.^[61]^

The activation of the Phosphotylinosital 3 kinase (PI3K)/ Protein kinase B (AKT) pathway and suppression of the neurogenic locus notch homolog protein (*NOTCH*) signaling pathway were believed to be potential prerequisites for SCLC transformation (Figure 2).^[61]^ The pharmacologic inhibition of the PI3K/AKT pathway exhibited promising results in suppressing tumor growth and preventing neuroendocrine transformation in a preclinical model using EGFR-mutant patient-derived xenografts.^[61]^ The *NOTCH*-ASCL1-*RB1*-*TP53* signaling axis has been identified as a potent pathway driving the pathogenesis of secondary SCLC. This pathway may be involved in the mechanism of SCLC transformation in cases where *RB1* deficiency is present.^[69]^ Several studies suggested that *RB1* deficiency was a necessary but not sufficient event leading to SCLC transformation. Animal models have provided evidence supporting the role of *RB1* loss in selectively affecting tumors of neuroendocrine origin, which supported the similarity between LCNECs and SCLC.^[70]^ Recent data has revealed that high levels of mitogen-activated protein kinase (MAPK) activation may prevent ADC from adopting a neuroendocrine identity. This finding suggested that the MAPK signaling pathway reaction could limit access to genes for neuroendocrine transcription factors, leading to certain SCLC cells transitioning into ADC.^[71]^ Therefore, the role of the MAPK signaling pathway in SCLC transformation is an area of promising future research. K. Ishioka *et al*. demonstrated that the upregulation of fibroblast growth factor 9 (FGF9) in established ADC cells has a context-dependent effect.^[72]^ Preclinical study conducted by Ferone *et al*. observed that FGFR1^K656E^ and *TP53*^F/F^ can modify the propensity of specific cell types to give rise to SCLC.^[73]^ In an *in vivo* study, transformed SCLC-like tumors showed a successful response to the pan-FGFR inhibitor AZD4547. This finding proposed that the FGF9-FGFR axis could be a potential therapeutic target for SCLC transformation.^[72]^

Several findings have provided valuable insights into the underlying molecular features involved in SCLC transformation. *BCL-2* has been identified as a possible key molecule in SCLC transformation.^[74]^ In a case report, *BCL-2* were found to be overexpressed in ADC with neuroendocrine transformation compared to the initial ADC.^[74]^ Similarly, Niederst *et al*. discovered several features of SCLC transformation, including activating mutation in *PIK3CA*, inactivation of *TP53* and sensitivity to *BCL-2* inhibition.^[52]^ Researchers also mentioned that ABT-263 (Navitoclax), a drug targeting *BCL-2* and *BCL-XL* inhibitors, exhibited marked efficacy against SCLC transformation from EGFR-mutant cell lines. However, ABT-263 failed to induce a robust apoptotic response in resistant EGFR mutant NSCLC cell lines harboring the T790M resistance mutation, and the mechanisms behind this failure remain unknown.^[52]^ Additionally, a study conducted by Gardner *et al*. highlighted the role of Schlafen family member 11 (*SLFN_11_*) suppression in acquired chemoresistance, which can be regulated by Enhancer of zeste homolog 2 (*EZH_2_*). This finding suggested that *EZH_2_* inhibitors, when combined with standard cytotoxic therapies, could potentially restore or maintain chemotherapeutic efficacy.^[75]^ Furthermore, *EZH_2_* inhibitors have shown promise in reversing lineage transformation and treating transformed SCLC patients.^[76]^ Other changes such as preferential APOBEC, which is rarely observed in EGFR T790M-positive ADC, can induce hypermutation and promote SCLC transformation through genomic instability.^[36]^ It is worth noting that previous studies have proposed several possible mechanisms driving the development of SCLC, including *TP53* mutation, *MYC* amplification (a known oncogenic driver for SCLC), and *PIK3CB* amplification.^[77,78]^ Recent studies have highlighted the potential therapeutic targeting of the nuclear transport protein Exportin 1.^[79]^ Inhibition of Exportin 1 has shown promise in preventing neuroendocrine transformation in ADC and prostate adenocarcinoma, thereby limiting the formation of invasive tumors. Researchers have discovered that the inhibition of Exportin 1 effectively suppresses the upregulation of the transcription factor sex determining region Y-box 2(SOX2), which plays a critical role in neuroendocrine transformation and is overexpressed in SCLC transformation.^[79]^ Additionally, it has been observed to enhance the effectiveness of standard chemotherapy in treating neuroendocrine tumors following histological transformation.^[79]^

Numerous strategies are currently under investigation to overcome acquired resistance resulting from SCLC transformation. The combination of cytotoxic chemotherapy and EGFR-TKIs has emerged as a potential first-line treatment option after SCLC transformation, demonstrating improved Progression-Free Survival (PFS) compared to mono-chemotherapy. Notably, the utilization of anti-angiogenic therapies and local radiotherapy has shown promising results in extending overall survival (OS) in these patients.^[80]^ Additionally, targeting aurora kinases (AURKA) inhibitors has shown potential in suppressing the growth of *Rb1*-negative SCLC cells, making it an attractive therapeutic approach for transformed SCLC patients. Furthermore, the inhibition of checkpoint kinase 1 (CHK1) and polo-like kinase 1 (PLK1), which target DNA damage checkpoints, holds promise as a therapeutic option.^[81,82]^ Moving forward, further research is needed to explore the efficacy and safety of these treatment strategies in larger clinical trials. Additionally, the identification of novel targets and the development of personalized therapies based on the molecular characteristics of transformed SCLC may pave the way for more effective and tailored treatment approaches in the future.

#### SCC transformation

Several cases of ADC with SCC transformation under treatment pressure have been reported. Adeno-squamous cell carcinoma (ASC), a subtype of NSCLC that consists of both adenomatous and squamous components in a single lesion, has been identified in approximately 4% to 9% of cases. Clinically, ASC is defined as a tumor with combined histological components exceeding 10%.^[59]^ Compared to either ADC or SCC, ASC has been reported as the most lethal form of NSCLC, with an unfavorable clinical prognosis.^[83]^

With the advancements in whole-exome sequencing (WES), it is now possible to identify the genomic landscape of SCC transformation. Clinical evidence demonstrated that ADC and transformed SCC shared the common driver genes.^[60,84,85,86]^ The loss of pre-existing or acquired mutations in ADC might trigger SCC transformation.^[87]^ Alvaro *et al*. observed that 85% of pre-transformation ADC cases lost the 3p chromosome arm in ADC to SCC transition cases, which was considered a risk factor for SCC transformation.^[61]^ However, no limited data from clinical trial study underscores the need for more extensive research on the origins of ADC to SCC transition.

Animal studies showed that ADC mice with *LKB1* deficiency have experienced SCC transformation.^[58,88]^ Understanding the downstream signaling pathways based on *LKB1* deficiency may help to find a therapeutic window for patients. The loss of *LKB1* leads to deregulation of the AMP-activated protein kinase-acetyl-coenzyme A carboxylase (AMPK-ACC) axis and deficiency of fatty acid oxidation (FAO) signaling pathway, resulting in increased reactive oxygen species (ROS) levels in lung ADC. This increase in ROS is also a consequence of decreased nicotinamide adenine dinucleotide phosphate (NADPH) levels and a dysregulated pentose phosphate pathway (PPP), particularly under conditions of nutrient scarcity that occur alongside tumor progression. The accumulation of excessive ROS causes a redox imbalance and disrupts the activation of downstream signaling molecules Nkx homeobox-1 gene (NKX2–1) and the forkhead box A2 (FOXA2). Meanwhile, *LKB1* loss leads to the degradation of lectin-like oxidized low-density lipoprotein oxidase (LOX) *via* the mammalian target of rapamycin-hypoxia-inducible factor 1α (mTOR-HIF1α) pathway. This event results in remodeling of the extracellular matrix (ECM), collagen deposition, the inactivation of yes-associated protein (YAP), and downregulation of zinc finger E-boxbinding homeobox 2 (ZEB2), ultimately, repressing TP63 (a predictor of SCC) transcription.^[59,89]^ YAP, a key downstream effector of the Hippo pathway, can significantly reverse the lung adeno-to-squamous transition phenotypic transition when activated.^[90]^ Researchers have reported that digitoxin, a compound that increases YAP activity, effectively suppresses the growth of SCC by targeting the WW domain of YAP.^[90]^ They also discovered that the knockdown of either large tumor suppressor 1 (*LATS1*) or the mammalian Ste20-like kinase1 (*MST1*), which are upstream negative regulators of YAP, adversely affects SCC development.^[90]^ Moreover, the activation of YAP resulted from digitoxin treatment, can alter the protein levels and subcellular distribution of glutathione peroxidase 2 (GPX2) expression, a direct target of TP63, disrupting ROS homeostasis.^[91]^ In a cell experiment study, the cell line with the highest EGFR expression among all SCC and ADC cell lines was identified. The results revealed that EGFR activation, whether through overexpression, amplification, or mutation, can lead to the activation of Yes-associated protein/Transcriptional co-activator with PDZ-binding motif (YAP/TAZ) *via* the phosphorylation of MOB kinase activator 1 (MOB1) and the concurrent inactivation of LATS1/2. This process occurs independently of the traditional Hippo pathway or MST1/2 kinases.^[92]^ Another study highlighted a positive signaling pathway loop involving EGFR, various EGF-like ligands, and the active YAP, which collectively contribute to the initiation and progression of SCC.^[93]^ These studies illustrated the importance of EGFR-MOB1-YAP/TAZ signaling axis in SCLC progression. Additionally, early clinical evidence has shown that *LKB1* loss leads to the activation of the PI3K/AKT signaling pathway.^[94]^ Inhibition of this pathway has been observed to re-sensitize imatinib-resistant, squamous-like tumors.^[42]^

Overall, the Hippo-YAP pathway, the EGFR-MOB1-YAP/TAZ signaling axis, and the PI3K/AKT pathway are complex and critical to SCC transformation. Targeted therapies that modulate these pathways warrant further investigation in clinical trials to establish their efficacy and potential as treatments for SCC (Table 1).

**Table 1:** Potential therapies or therapeutic target for histological transformation in ADC

Histological subtypes	Existing pathways and potential therapies	References
Small cell lung carcinoma	Combinational therapy of cytotoxic chemotherapy and TKIs	[80]
	BCL-2 inhibitors, Exportin 1 inhibitors, CHK1 inhibitors, PLK1 inhibitors, AURKA inhibitors	[52,74,79,81,82]
	Targeted therapies against FGF9-FGFR axis, EZH_2_-SLFN_11_ Axis, NOTCH signaling pathway, PI3K-AKT pathway	[61,69,72,73,75,76]
	Activation of MAPK signaling pathway	[71]
Squamous cell carcinoma	Targeted therapies to overcome dependencies on Hippo YAP pathway, EGFR-MOB1-YAP/TAZ signaling axis, PI3K-AKT pathway	[42]
Sarcomatoid carcinoma	Targeted therapies against EMT pathway	[99,100]
	PD-1/PD-L1 inhibitors, MET inhibitors	[101]
Large cell neuroendocrine carcinoma	Combinational therapy of immune checkpoint inhibitor and chemotherapy	[104,106]

ADC: lung adenocarcinoma; TKIs: Tyrosine Kinase Inhibitors; BCL-2: B-cell lymphoma 2; CHK1: checkpoint kinase 1; PLK1: polo-like kinase 1; AURKA: aurorakinase A; FGF9: fibroblast growth factor 9; FGFR: fibroblast growth factor receptor 1; EZH_2_: Enhancer of zeste homolog 2; SLFN_11_: Schlafen family member 11; NOTCH: neurogenic locus notch homolog protein; PI3K: Phosphatidylinositol-3-kinase; AKT: Protein kinase B; EMT: epithelial-mesenchymal transition; PD-1/PD-L1: programmed cell death 1/programmed death ligand 1; MET: cellular-mesenchymal to epithelial transition.

### Pulmonary sarcomatoid carcinoma transformation

Pulmonary sarcomatoid carcinoma (PSC) transformation is one of the resistant mechanisms of ADC. Although it is reported less frequently compared to other types of histologic transformation, the mechanisms of PSC transformation are complexed.^[95]^ Unlike other lung cancer transformation that directly transformed from ADC, PSC transformation can have multiple steps. A case study reported a patient with EGFR-mutated ADC first transformed to SCC, then subsequently to PSC after treatments.^[95]^ Lee supposed that this event resulted from intra-tumor heterogeneity.^[95]^ It is worth to note that, in this study, Lee and his coworkers also observed additional acquired mutation in transformed tumor. After developing to SCC transformation, the EGFR mutation assay of this patient showed an exon 19 deletion and T790M three years and 9 months after osimertinib treatment. However, rebiopsy showed PSC transformation after 11months later, and EGFR assay showed only exon 19 deletion. The loss of T790M mutation was the resistance mechanism to osimertinib.^[95]^ In line with this study, another case showed the similar phenomenon. Li *et al*. reported an EGFR-mutated ADC patient who experienced PSC transformation in a metastatic lung region, followed by neuroendocrine transformation from ADC in liver metastatic lesion after TKIs therapies.^[96]^ Through driver gene alteration testing, multiple gene mutations were identified at different stages of treatment. These included the discovery of EGFR amplification and T790M after erlotinib resistance, as well as the identification of *AKT1 E17K* and a large fragment deletion of *RB1* after osimertinib resistance. However, only EGFR amplification and a large fragment deletion of *RB1* were consistently observed in all transformed tumors. Notably, after using combination therapy targeting EGFR amplification, the patient achieved some clinical benefit.^[96]^ Li *et al*. proposed a possible explanation, suggesting the importance of trunk clone evaluation and tumor heterogeneity. They postulated that under drug selective pressure, EGFR amplification initially emerged as the trunk mutation. Subsequent resistant mechanisms, such as the large fragment deletion of *RB1* and histological transformation, were considered as divergent propagation of subclones originating from the EGFR amplification. Li *et al*. emphasized the importance of targeting the major clone of the tumor and making timely adjustments to therapeutic approaches based on the dynamic changes in genetic characteristics during treatment.^[96]^ This highlights the need for a personalized and adaptable treatment strategy in order to effectively address the evolving nature of the tumor. Similarly, the coexistence of PSC transformation and oncogenic drug-resistant mutations can also occur in non-EGFR mutations. Scientists reported a patient with *ROS* Proto-Oncogene 1, Receptor Tyrosine Kinase (*ROS1)* rearrangement ADC who experienced PSC transformation later. The results of next-generation sequencing showed *CD74^-^ROS1* fusion and *ROS1-F2004C* mutation after transformation.^[16]^ These studies highlighted that histological transformation and other acquired mutations may coexist during disease progression and serve as resistant mechanisms.^[16,95,97,98]^

Previous studies suggested that PSC transformation could be interpreted as a kind of epithelial-mesenchymal transition (EMT). Jiang *et al*. reported a patient with SCC who experienced crizotinib-resistant morphologic PSC transformation afterwards. In samples of this patient, the expression of vimentin was positive, which revealed the possibility of EMT development.^[99]^ Another multi-omics analysis revealed that EMT played an important role in epithelial-to-sarcomatoid components transformation in PSC.^[100]^ Therefore, EMT could be a potential therapeutic target for PSC in the future. Additionally, aberrant activation of cellular-mesenchymal to epithelial transition (MET) factor and programmed death-ligand 1 (PD-L1) were also associated with sarcomatoid transformation. Hsieh speculated that MET copy number and PD-L1 expression could be indicators of PSC transformation.^[101]^ Yet, more clinical works are still warranted to determine more specific mechanisms.

### LCNEC transformation

As a high-grade neuroendocrine carcinoma, LCNEC transformation shared similar clinical features and genomic characteristics with SCLC transformation.^[36]^ Previous studies suggested that the underlying molecules mechanisms of both SCLC and LCNEC transformation may be the loss of *Rb1*/*TP53*.^[63,102,103]^ Two cases reported osimeitinib-resistant T790M negative LCNEC transformation from T790M positive ADC.^[104,105]^ Both of them suggested that T790M negative mutation was acquired on-target resistance to osimertinib. One of the two cases considered the mechanisms of LCNEC transformation may be inactivation of *TP53* and *Rb1*, which were similar to SCLC transformation.^[104]^ Moreover, Shinichi *et al*. pointed out that EGFR T790M-positive ADC had rare APOBEC-associated mutation, which can promote SCLC transformation. Thus, they inferred that EGFR T790M-positive ADC may be indirectly related to LCNEC transformation.^[104]^ Generally, although LCNEC was resistant to chemotherapy, early evidence proved that subsequent carboplatin combined with etoposide therapy after osimertinib-resistant transformed LCNEC was efficient, which raising hopes for more personalized treatment.^[104,106]^ However, little known about potential mechanisms and treatments have been proposed, as cases of LCNEC transformation were rare. More research should be conducted.

## Summary and future perspective

To sustain the momentum of discovery and improve prognosis in patients with transformed lung cancer, several keys lie ahead. Previous studies focused on approved therapies for those patients, with continued exposure to systemic agents, cancers typically become heterogeneity and genomic complexity. Heterogeneity can develop and can undermine the therapeutic efficacy of potent and selective TKIs. Consequently, the efficacy of subsequent lines of therapy failed to improve outcomes. Upfront treatment with potent pan-inhibitory TKIs can be more effective than delaying the utilization of these agents until the second-line and beyond, although it is impossible to completely eradicate resistant clones. Instead, treatment with more potent TKIs may alter the trajectory of clonal evolution. A phase III study implementing upfront second-generation ALK TKI alectinib in ALK-positive NSCLC demonstrated a 7% improvement in response rates.^[107]^ Additionally, the combination of therapies targeting different mechanisms (*e.g*., chemotherapy/immunotherapy/ antibody-drug conjugates [ADC] and TKIs) is predicted to be beneficial in tumors with high intra-tumor heterogeneity.^[108,109,110]^ Recent experiments revealed encouraged outcomes of all-trans retinoic acid (ATRA). ATRA can prevent TKI-induced enrichment of CSCs. The combination of ATRA and platinum-based chemotherapy can prolong disease control in LCNEC and SCLC.^[111,112,113]^ Current study mentioned that Selinexor (an inhibitor of Exportin 1) can inhibit the activation of the tumor AKT signaling pathway induced by chemotherapy. Researchers further discovered that selinexor combined with chemotherapy can suppress the growth of transformed SCLC cells.^[79]^ However, no sufficient clinical trials were conducted to optimize treatments for lung cancer transformation patients.

Besides, the screening and development strategies for patients with high-risk of histological transformation remains a huge barrier. Identification of precise biomarkers are challenging but necessary tasks in predicting transformation and guiding the next treatment. Several evidence suggested that the detection of plasma pro-gastrin-releasing peptide (Pro-GRP), neuron-specific enolase (NSE), and cytokeratin-19-fragment (CYFRA21–1) can identify patients with ADC at a high-risk of SCLC transformation and LCNEC transformation.^[114,115,116]^ Kato *et al*. reported increased level of ProGRP before re-biopsy-confirmed SCLC transformation.^[114]^ Jin *et al*. detected markedly elevated NSE levels in six cases at the time of SCLC transformation.^[117]^ Moreover, TP53 and Rb1 loss has been identified as dangerous signs of ADC to SCLC transformation.^[10,81,118,119,120]^ However, Rb1 loss alone is necessary but insufficient for fully penetrant transformation.^[56]^

Preliminary outcomes revealed that molecules including MET and PD-L1, which present as baseline, can be predictors of PSC transformation.^[95,100]^ Additionally, vimentin overactivation can promote an EMT-phenotype similar to that reported for the sarcoma subtype. Thus, vimentin have the possibility of indicating histological transformation mediated by EMT.^[99]^

It is far enough to characterize histological transformation by pathological diagnosis solely. Liquid biopsy offers possibility to detect and monitor different cancers in an easily accessible modality. ctDNA can discover the gene alterations of tumors, which may help identify patients who are at high-risk of progressing SCLC transformation and will benefit from tissue biopsy.^[121,122]^ With the manifestation of real-time and application of cytopathological analysis, circulating tumor cells (CTCs) detection by morphology-based enrichment methods may be applied to monitor the clonal evolution of solid tumors and perform molecular features.^[123,124]^ Zhu *et al*. developed an aptamer-modified PEG-PLGA-nanofiber (PPN) microfluidic system optimized for recognizing rare CTC subtypes in lung cancer patients. When combined with downstream single-cell sequencing, the aptamer-modified-PPN microfluidic system can assess tumor heterogeneity and predict histological transformation.^[15]^ Thus, it may be a promising substitute for tissue rebiopsy to detect histologic transformation.

In summary, histological transformation reflects the strong plasticity of lung cancer. Integrative studies of murine models and human clinical specimens have convincingly corroborated that histological transformation was an emerging and important mechanism of treatment resistance. The most plausible hypothesis is branch evolution in common cellular origin. Possible pathophysiological mechanisms, such as heterogeneity of tumor, might be involved in underlying the development of histological transformation. Moving forward, the different mechanisms of signaling pathways in tumors with histologic transformation potentially be a breakthrough in developing novel biomarkers and therapies. We encourage the exploration of clinical trial options whenever possible, including newly diagnosed patients.
